# Longitudinal Optical Coherence Tomographic follow-up of a 10-year-old girl with unilateral retinal parasitism

**DOI:** 10.22336/rjo.2024.59

**Published:** 2024

**Authors:** Omer Karti, Turhan Mammadov, Songul Bayram Delibas, Ali Osman Saatci

**Affiliations:** 1 Department of Ophthalmology, Dokuz Eylul University, Izmir, Turkey; 2 Department of Parasitology, Dokuz Eylul University, Izmir, Turkey

**Keywords:** nematodes, ocular parasitosis, optical coherence tomography, retinal helminthic infections, zoonotic parasitic disease, OCT = Optical Coherence Tomography, SD-OCT = spectral domain optical coherence tomography

## Abstract

**Objective:**

Retinal parasitism by worms is a rare clinical occurrence and may cause diagnostic and therapeutic challenges. We present a girl with unilateral involvement who was first diagnosed at the age of 10. Change in parasite appearance inside the lesion was recorded by optical coherence tomography (OCT) 14 years apart.

**Methods:**

Case report.

**Results:**

A 10-year-old girl was referred to our clinic with a presumptive diagnosis of a unilateral posterior pole mass. She had been examined at another institution for the left exotropia and decreased vision, without any accompanying systemic symptoms. Upon our examination, a subretinal lesion, of two disc diameters in size, was detected two disc diameters above the left optic disc, and a spiral-shaped, non-motile roundworm could be spotted inside the lesion. Adjacent chorioretinal atrophy and marked fibrosis surrounded the lesion, without any active inflammation. OCT sections through the lesion delineated the hyperreflective appearance of the spiraling roundworm. Fourteen years later, the worm inside the lesion had lost its spiraling form, and the remnants appeared as a coalesced whitish material inside the lesion on the OCT.

**Discussion:**

Although rare, retinal parasitism by worms presents significant diagnostic challenges due to its atypical presentation and the potential for misdiagnosis. In this case, the initial presentation of a subretinal lesion containing a spiral-shaped roundworm was notable for its lack of associated systemic symptoms and the absence of active inflammation, often seen in more common ocular infections. The long-term follow-up, using OCT, provided valuable insights into the infection’s natural course, showing the gradual degeneration and transformation of the parasite into a coalesced whitish material, over 14 years. This case underlines the importance of longitudinal imaging in understanding the progression of such unusual retinal conditions and the need for awareness of parasitic infections as a differential diagnosis in similar clinical scenarios.

**Conclusions:**

The present case demonstrates the natural evolution of the inactive subretinal worm by OCT and color fundus images.

## Introduction

Systemic parasitic infections are prevalent world wide, with certain types capable of affecting the ocular tissue involving both the anterior and/or posterior segments. Many parasitic infections are uncommon in temperate climates, leading to unfamiliarity among ophthalmologists practicing in those areas. Conversely, clinicians residing in tropical locations are often very familiar with these parasitic diseases [1]. The prevalence of these infections varies depending on factors such as geographic location, host hygiene, cultural practices including living conditions and dietary habits, and the proximity to the animals [2-4]. The causative parasites may vary from simple, single-celled protozoans, to complex metazoan helminths, including nematodes, cestodes, and trematodes [3]. Herein, we present a case with a unilateral, subretinally located unidentified helminth causing severe fibrosis and surrounding chorioretinal atrophy.

We followed the evolution of the worm longitudinally by spectral domain optical coherence tomography (SD-OCT) and color fundus images.

## Case report

**Fig. 1:**
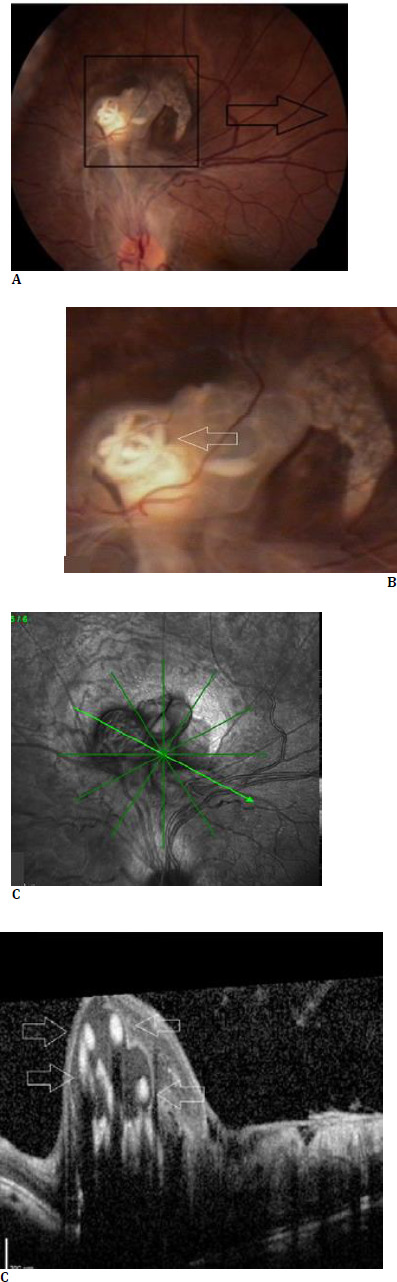
Left eye at the presentation in 2010. Color fundus pictures (**A** and **B**) showing the two-disc-diameter-sized subretinal lesion housing a spiral-shaped non-motile roundworm (white arrow) located two disc diameters above the optic disc. Marked fibrosis surrounded the lesion, without any active inflammation and adjacent chorioretinal atrophy. Optical coherence tomographic section (**C**) through the lesion revealed the hyperreflective spiraling, round-shaped subretinal parasite (white arrows), with posterior shadowing

**Fig. 2:**
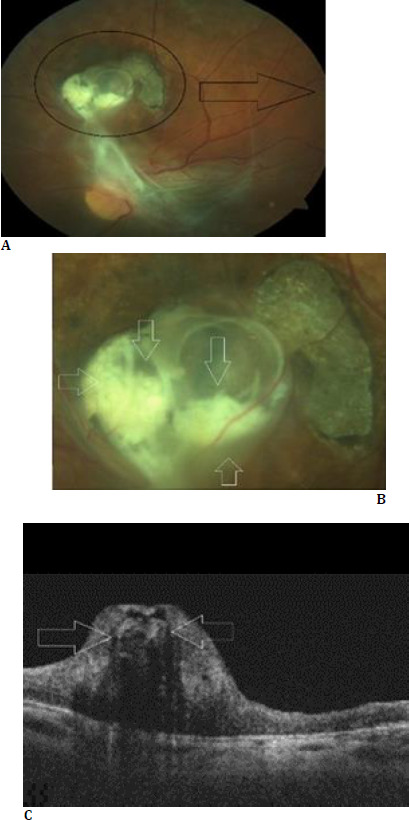
Left eye at the final visit in 2024. Color fundus pictures (**A** and **B**) demonstrate the unchanged size of the retinal lesion, with degradation of the worm within (white arrows). The presence of epiretinal membrane was more evident. The worm inside the lesion had lost its spiraling form, and the remnants appeared as coalesced whitish material (white arrows) inside the lesion on the optical coherence tomography (**C**)

A 10-year-old otherwise healthy girl was referred to our clinic with a presumptive diagnosis of a retinal mass for a second opinion. She had left exotropia and decreased vision, without any accompanying systemic symptoms. The patient lived in Aydin province, in the Aegean region, presenting no prior international or domestic travel. Her socioeconomic status was not low and she had standard hygiene conditions. No history of insect bites or eventful animal encounters and dietary habits, including consuming raw meat products, were present. On our examination, she had left exotropia with full motility. The best-corrected visual acuity was 20/20 in the right eye and 20/200 in the left eye. Slit-lamp examination was unremarkable bilaterally. Intraocular pressures were within normal limits in both eyes. While the right fundus was normal there was a two-disc-diametersized subretinal lesion housing a spiral-shaped nonmotile roundworm inside, located two disc diameters above the left optic disc. Marked fibrosis, surrounding the lesion without any active inflammation, and adjacent chorioretinal atrophy were present (**Fig. 1 A, B**). OCT scan passing through the lesion revealed the hyperreflective spiraling, round-shaped subretinal parasite, with posterior shadowing (**Fig. 1 C**). Full biochemistry, chest X-ray, urine, and stool analysis turned negative. The Eliza test for toxocara was negative. Color fundus pictures were reviewed with a senior parasitologist to guess the causative agent. Precise determination of the causative agent could not be done but the suspicion was leaned towards a nematode. The patient was monitored without intervention. Fourteen years later, the size of the lesion looked unchanged but there was some degradation of the worm within. Additionally, the epiretinal membrane, extending inferiorly from the lesion area, looked more evident (**Fig. 2 A, B**). The worm inside the lesion had lost its spiraling form, and the remnants appeared as a coalesced whitish material inside the lesion on the OCT (**Fig. 2 C**). An attempt was made to obtain the patient’s intact OCT images. However, an assessable image could not be obtained due to significant motion artifacts. Given the stability of the patient’s vision and the absence of ocular inflammation, no further intervention was recommended.

## Discussions

Zoonotic helminths can be located in the vitreous, retina, and/or choroid, leading to various ocular complications, including retinal hemorrhage (caused by Ancylostoma, Gnathostoma, Toxocara, Trichinella), retinal detachment (associated with Taenia), intraretinal cysts (linked to Echinococcus), and retinitis/choroiditis (caused by Baylisascaris, Taenia, Toxocara, Trichinella, Onchocerca) [5]. These conditions can result in severe vision loss [5], not primarily due to scarcity of treatment options, but largely because of the delayed or incorrect diagnosis, often stemming from unfamiliarity with the disease course [3]. In our case, a helminth was discovered within the subretinal space, leading to strabismus and severe vision loss. It was incidentally detected during a dilated fundus examination when the family noticed the squint. The patient lived in Aydin province, in the Aegean region, presenting no prior international or domestic travel. As far as we know, no helminth discovered within the retina has been previously described as coming from this region. However, Ilhan et al. [6] reported a case involving a painless, immobile mass beneath the conjunctiva in a 28-year-old otherwise healthy man residing in Aydin. Upon tissue surgical removal, they identified a filarial nematode infection (Onchocerca lupi). When reviewing the literature, no data regarding the retinal involvement of this parasite could be found.

Ocular toxocariasis could be in the differential diagnosis and this helminthic infection seems to be prevalent in Turkey with reported seroprevalence rates ranging between 4% and 64% [7]. Three main patterns of ocular toxocariasis have been described: posterior granuloma typically found submacularly or peripapillary and characterized by pronounced retinal striae or folds; peripheral granuloma, often accompanied by a vitreoretinal traction band extending towards the posterior pole; and severe, unilateral intermediate or panuveitis [8]. The immune response occurs to contain Toxocara larvae and thereby results in granuloma formation and thereby parasite isolation can occur. Posteriorly located granulomas typically affect individuals aged 6 to 15 years. These patients manifest as a solitary yellow-white mass, ranging from 0.5 to 3 disc diameters in size, located at the posterior pole. Minimal or no intraocular inflammation is usually present. The lesion is typically well-defined and situated within the subretinal space, often accompanied by tractional bands between the granuloma and the surrounding retina [9]. A moderately hyper-reflective retinal round mass, sometimes exhibiting posterior shadowing, is defined as the OCT finding of toxocara granuloma. These masses remarkably distort the retina and may be accompanied by epiretinal membrane formation and photoreceptor disruption [10]. However, the retinal lesion in the present case was incompatible with classical toxocara infection. Repeated OCT examinations 14 years apart showed the evolution of the parasite in the present case. Therefore, we thought that the helminth in our case might be a different parasite. In the present case, the follow-ups with OCT were very useful in observing the parasite’s evolution.

The causative agent diagnosis typically requires the worm’s surgical removal and, thereby, the examination of its microscopic features. Surgery serves, not only as a diagnostic measure but can also be curative in some instances. In the present case, we chose not to intervene surgically due to exotropia, with possible amblyopia and quiet-looking fundus. Unfortunately, the naming of the helminth could not be done.

## Conclusion

The present case demonstrates the natural evolution of a subretinal worm with the help of OCT and color fundus images taken 14 years apart. Longitudinal fundus examinations and OCT images have shown that helminths in the retina can remain inactive for years, with natural degradation of the parasite over time. Our case report highlighted the importance of documenting and multimodal imaging. Prudent follow-up can be a logical option in stable cases without any signs of inflammation.
